# Mindfulness-Based Interventions for Adolescent Social Anxiety: A Unique Convergence of Factors

**DOI:** 10.3389/fpsyg.2020.01783

**Published:** 2020-07-22

**Authors:** Corinne N. Carlton, Holly Sullivan-Toole, Marlene V. Strege, Thomas H. Ollendick, John A. Richey

**Affiliations:** ^1^ Clinical Science Program, Department of Psychology, Virginia Tech, Blacksburg, VA, United States; ^2^ Graduate Program in Translational Biology, Medicine, and Health, Virginia Tech, Blacksburg, VA, United States

**Keywords:** mindfulness-based interventions, social anxiety, adolescent, anxiety, mindfulness

## Abstract

Social anxiety disorder (SAD) is a debilitating and often chronic psychiatric disorder that typically onsets during early adolescence. Cognitive behavior therapy (CBT), the current “gold-standard” treatment for SAD, tends to focus on threat- and fear-based systems hypothesized to maintain the disorder. Despite this targeted approach, SAD ranks among the least responsive anxiety disorders to CBT in adolescent samples, with a considerable proportion of individuals still reporting clinically significant symptoms following treatment, suggesting that the CBT-family of interventions may not fully target precipitating or maintaining factors of the disorder. This gap in efficacy highlights the need to consider new therapeutic modalities. Accordingly, this brief review critically evaluates the emergent literature supporting the use of mindfulness-based interventions (MBIs) for treating adolescent SAD. MBIs may be particularly relevant for addressing maintaining factors within this diagnosis, as they may target and interrupt cycles of avoidance and de-motivation. Despite limitations in the relative lack of randomized controlled trials (RCTs) on this topic, a unique convergence of factors emerge from the extant literature that support the notion that MBIs may hold particular promise for attenuating symptoms of SAD in adolescents. These factors include: (1) MBIs demonstrate the ability to directly engage symptoms of SAD; (2) MBIs also show consistent reduction of anxiety, including symptoms of social anxiety in adolescent populations; and (3) MBIs demonstrate high rates of feasibility and acceptability in anxious adolescent samples. We briefly review each topic and conclude that MBIs are an encouraging treatment approach for reducing symptoms of social anxiety in adolescents. However, given the lack of research within MBIs for adolescent SAD in particular, more research is needed to determine if MBIs are more advantageous than other current treatment approaches.

## Introduction

Social anxiety disorder (SAD) is a common and functionally impairing psychiatric disorder marked by fear of one or more social or performance situations (American Psychiatric Association, 2013), and that follows a chronic and generally unremitting course throughout the lifespan if left untreated (Albano and Hayward, 2004; Knappe et al., 2015). Despite considerable progress in prevention and intervention efforts, SAD remains among the most common anxiety disorders in both adolescents and adults, with an estimated lifetime prevalence rate of approximately 12% in the United States (Kessler et al., 2005; Beesdo et al., 2007; Knappe et al., 2015). Moreover, SAD is associated with a significantly diminished quality of life (Saarni et al., 2007; Aderka et al., 2012) and substantial functional impairment across a variety of contexts including interpersonal, educational, and occupational domains (Acarturk et al., 2008). SAD typically emerges in early adolescence, around roughly 13–14 years of age (Ollendick and Hirshfeld-Becker, 2002; Kessler et al., 2005; Farrell et al., 2019). Among adolescents, prevalence rates for SAD are between 10 and 15% (Essau et al., 1999; Heimberg et al., 2000; Merikangas et al., 2010), ranking it among the most common anxiety disorders during this developmental period (Merikangas et al., 2010). Cognitive behavior therapy (CBT) for SAD is currently considered the “gold-standard” treatment for both adults and adolescents (Gordon et al., 2014). Insofar as psychological interventions tend to be most effective when administered at or around the age of onset (Spence et al., 2000; Herbert et al., 2009; McGorry et al., 2011), CBT for socially anxious adolescents should be particularly effective when delivered in this age range. To the contrary however, SAD remains among the least treatment-responsive disorders to CBT in adolescent samples (Hudson et al., 2015), and is effective in only 40–65% of cases (Ginsburg et al., 2011). These relatively modest rates of success suggest that current approaches in the CBT-family of interventions may not comprehensively target precipitating or maintaining factors, thus highlighting a critical need to consider new treatment modalities that may hold promise for affected individuals.

Accordingly, this brief review focuses on the potential for mindfulness-based interventions (MBIs) to specifically modulate SAD symptomology in adolescents. Through this review, we describe a unique convergence of factors that highlight the potential utility of treating adolescents with SAD with MBIs. First, we discuss MBIs and their background and relevance to social anxiety. Next, we describe preliminary evidence for the efficacy of MBIs in adolescent populations. Finally, we discuss the feasibility and acceptability of MBIs for adolescents. This prior work has demonstrated that (1) MBIs directly engage symptoms of SAD; (2) MBIs show consistent reduction of anxiety in adolescent populations; and (3) MBIs demonstrate high rates of feasibility and acceptability in anxious adolescent samples; making MBIs a potentially viable approach for treating adolescent SAD. However, although MBIs may be particularly promising for adolescent SAD, the current literature base in this area is in its infancy; warranting more research in this area.

## Mindfulness-Based Interventions: Background and Relevance to Social Anxiety

Mindfulness can be defined as the awareness that arises when paying attention in the present moment, on purpose and non-judgmentally (Kabat-Zinn, 1990). When someone is “being mindful,” they adopt the attitudinal quality of not judging and allowing experience to unfold with curiosity rather than trying to manage or control it. This approach may reduce the impact of positive and negative affective states when triggered (Brewer et al., 2015). Two well-established MBIs include mindfulness-based stress reduction (MBSR) and mindfulness-based cognitive therapy (MBCT). A central premise in both MBSR and MBCT is experiential instruction in observing thoughts, feelings, and physical sensations in the present moment with an attitude of non-judgmental acceptance. A primary objective of MBIs in this context is to enhance identification of negative and ruminative thoughts as well as their triggers, and also to promote a shift in perspective such that these responses can be seen as mental events rather than representations of reality. A noteworthy difference between MBSR and MBCT is that MBCT incorporates didactic components focusing on CBT techniques (e.g., focus on identification of negative automatic thoughts), whereas MBSR does not (see Baer, 2003). Further, MBCT includes specific modules derived from core CBT tenets that were originally used to treat recurrent depression by targeting ruminative thought processes through increasing awareness and decreasing engagement in repetitive negative thinking about symptoms (Segal et al., 2002). Although no study has yet compared MBSR to MBCT to determine differential impacts on treatment outcomes in SAD, both approaches may be relevant to addressing maintaining factors within this diagnosis. For example, periods of transient social stress (including apprehension about future social events) activate negative and ruminative patterns of thinking, principally including anticipatory fear of negative evaluation (Mellings and Alden, 2000; Nolen-Hoeksema, 2000; Fresco et al., 2002; Harrington and Blankenship, 2002; Robinson and Alloy, 2003). Over time, these patterns of repetitive negative thinking lead to behavioral avoidance of feared situations and as a secondary effect may also uncouple the experience of social interaction from its normally rewarding consequences (Klemanski et al., 2017; Richey et al., 2019). By eroding motivation to pursue social interactions (including social behaviors with the potential for a gratifying or rewarding outcome; Insel, 2003; Ottenbreit et al., 2014), behavioral avoidance may also exacerbate social skills deficits observed in SAD. Mindfulness practice may interrupt this cycle of avoidance and de-motivation by promoting non-judgmental observation of thoughts, feelings, and sensations, thus recoupling social interaction behaviors with the experience of rewarding or otherwise gratifying outcomes that may result from socializing (Richey et al., 2019).

Although systematic work in the area of mindfulness training specifically for SAD in adolescence remains scarce, MBIs have a strong evidence base of attenuating SAD-related symptomology in adults (see review by Norton et al., 2015). Recent meta-analyses in adults with a variety of other anxiety disorders indicate that MBIs reliably reduce anxiety symptoms yielding effect sizes ranging from 0.30 to 1.0 (Hofmann et al., 2010; Khoury et al., 2013; Goyal et al., 2014). Prior evidence from randomized controlled trials (RCTs) in adults with SAD has consistently indicated comparable, although not superior treatment outcomes in MBIs as compared to CBT in adults (Kocovski et al., 2013; Goldin et al., 2016). However, there is no current research examining a comparison of efficacy rates between CBT and MBIs in adolescents with SAD. Although outcomes are comparable with CBT, MBIs target different mechanisms of change (e.g., positive emotionality and reward-based learning) that appear to be unique to SAD relative to other anxiety disorders and may lead to certain advantages in socially anxious adolescent populations (for full review, see Richey et al., 2019). Further, RCTs in adults with SAD have shown that treatment with MBIs resulted in increased mindfulness skills, social adjustment, self-compassion, attention regulation, self-esteem, better overall functioning, and quality of life (Goldin et al., 2009, 2013; Goldin and Gross, 2010; Cassin and Rector, 2011). Additionally, outcomes of MBI treatment have shown decreased symptoms of social anxiety, negative self-views, trait anxiety, negative emotional reactivity, and depression, as well as increased positive affect and positive self-view (Koszycki et al., 2007; Jazaieri et al., 2012; Goldin et al., 2013; Faucher et al., 2016). Further, a study by Piet et al. (2010) demonstrated that the effects of mindfulness interventions may be relatively long-lasting. They examined the impact of MBCT on young adults (i.e., 18–25 years old) with SAD and found that this mindfulness intervention actively reduced social anxiety at post-treatment, with further improvements at 6- and 12-month follow-up.

While social anxiety symptom improvement has been observed across these studies, each study investigated distinct hypothesized mechanisms of change (e.g., social adjustment and negative emotional reactivity). As a consequence, it is difficult to identify direct connections between existing clinical trials in adults and processes of change. One exception to this however is provided by a recent pilot study by Strege et al. (2018), who specifically probed hypotheses related to mechanisms of change in systems of positive emotionality. This study evaluated the impact of MBCT on dimensions of positive and negative affect in adults with SAD and a psychiatric comparison group of adults with generalized anxiety disorder. Both groups improved on overall measures of symptomatology, but results further suggested that mechanisms of change from mindfulness practice may be distinct between these two groups. In SAD specifically, changes in positive, approach-related emotion were demonstrated.

Further, outcomes from MBIs have been compared to those from CBT and other forms of treatment [e.g., aerobic exercise; cognitive behavioral group therapy (CGBT)], and have been shown to have both comparable outcomes and, in several instances, improved outcomes (Jazaieri et al., 2012; Goldin et al., 2016). However, the current status of literature is mixed as to whether MBIs hold a significant advantage over CBGT (Koszycki et al., 2007; Kocovski et al., 2013). Despite this, a study by Goldin et al. (2017) examined the trajectories of treatment outcomes in MBSR vs. CBGT and found that although there were similar rates of reductions in SAD diagnosis at post-treatment, MBSR elicited treatment advantages in both greater rates of acceptance of anxiety and acceptance success (i.e., perceived ability to successfully accept anxiety). Further, this study determined that individual variation in weekly mindful attitudes and the disputing of anxiety (i.e., challenging of anxious thoughts and feelings) were predictive in decreasing social anxiety symptomatology. Of note, while many other manualized CBT approaches exist, given space limitations we have limited our review to the strongest current evidence base. According to this work, the application of MBIs to socially anxious samples appears to have strong precedent.

## Preliminary Evidence of Efficacy for MBIs for Adolescent Psychiatric Symptoms and Social Anxiety

While the available evidence for the efficacy of MBIs for adults with SAD is encouraging, RCTs for socially anxious adolescents remain very few in number. A recent meta-analysis by Dunning et al. (2019) examined 33 studies and reported that MBIs held particular promise across multiple domains in adolescent populations, for example in increasing self-reported mindfulness (*d* = 0.42), and decreasing symptoms of depression (*d* = 0.47) and anxiety (*d* = 0.18). RCTs examining the efficacy of MBIs have utilized various MBI protocols (e.g., Taming the Adolescent Mind; MBSR) as well as heterogeneous psychiatric samples (Biegel et al., 2009; Tan and Martin, 2015; Díaz-González et al., 2018). For example, Biegel et al. (2009) examined the efficaciousness of MBSR in a large (*N* = 102) adolescent sample with diverse psychiatric symptoms. Participants were randomized to either MBSR or a waitlist control group. Participants assigned to MBSR participated in 2-h weekly group meetings over 8 weeks as an adjunct to the current psychological services they were receiving. Results indicated that in both the intent-to-treat and completer samples, the addition of MBSR resulted in a greater reduction in self-reported anxiety. It should be noted that this group received more treatment compared to the control group. However, these results support the notion that MBIs can reduce anxiety symptoms in this population.

In another RCT, Tan and Martin (2015) examined the efficaciousness of a different mindfulness-based group intervention “Taming the Adolescent Mind” (Tan and Martin, 2013), a 5-week protocol involving mindfulness psychoeducation and exercises adapted for an adolescent population (13–18 years old) from the MBSR protocol. Adolescents with diverse psychiatric symptoms from outpatient mental health clinics were randomly assigned to receive a mindfulness intervention as an adjunct to current therapy services or waitlist control. The researchers found that individuals in the adjunct mindfulness intervention group showed a greater improvement in mental health composite scores composed of self-reported anxiety, depression, and stress and parent-report of the child’s psychological functioning. An additional RCT by Diaz-González et al. (2018) examined the impact of MBSR on anxiety symptoms in adolescents (between the ages of 13–16 years old) being treated for various anxiety disorders. Adolescents were randomly assigned to one of two groups: MBSR plus treatment as usual or only treatment as usual. Results from this RCT indicated that adolescents in the MBSR condition showed significantly decreased anxiety symptoms. Collectively, these results suggest that treatment with MBIs reduces anxiety symptoms in adolescents, providing additional support for the efficacy of MBIs for SAD in adolescent populations.

Two recent uncontrolled trials have also found evidence for efficacy of MBIs among adolescents across a variety of anxiety disorders, including SAD. Cotton et al. (2016) utilized a MBCT child protocol based on Semple and Lee (2011) manualized approach in a small (*N* = 10) open clinical trial. Participants were children/adolescents with an anxiety disorder diagnosis, who also had a parent with bipolar disorder. The intervention involved 20 weekly sessions, and participants were separated into one of two age groups (9–12 and 13–16 years). Participants experienced a reduction in anxiety symptoms (both clinician- and self-report) from pre- to post-intervention. Cotton and colleagues further found that increases in mindfulness were related to reductions in anxiety symptoms (both clinician- and self-report). In a conceptually related study, Crowley et al. (2018) utilized group mindfulness therapy for anxiety specifically tailored to adolescents, in a small (*N* = 11), uncontrolled trial. Participants were 12–13 years old with elevated anxiety scores. The intervention involved 10 weekly sessions, including components of mindful breathing, walking, and eating, as well as body scans and loving-kindness practice. Improvements were found for both youth- and parent-reports of internalizing symptoms as well as youth-reports of anxiety and perceived stress, with effect sizes from 0.88 to 1.34.

Whereas both Cotton et al. (2016) and Crowley et al. (2018) reported data from adolescent samples with various anxiety diagnoses, in the only known trial to utilize an adolescent SAD sample specifically, Ebrahiminejad et al. (2016) conducted a small (*N* = 30), randomized controlled trial to examine the efficacy of MBIs in socially anxious adolescent females. The rationale motivating the use of MBI in this particular sample was that traditional means of treatment (i.e., CBT) were (1) too lengthy, (2) required a high-level of expertise in order to effectively implement, and (3) had “lower-than-expected” outcomes. Therefore, they sought to examine the effectiveness of MBCT on diminishing social anxiety symptoms and improving self-esteem in these adolescents. All participants also met a clinical cutoff on the social phobia inventory (SPIN; Conner et al., 2003). Participants were randomly assigned to either the treatment or control condition (no treatment). The intervention involved 8 weekly group sessions based on the MBCT protocol and home meditation and mindfulness exercises. Results indicated that individuals in the MBCT group showed a significant reduction in self-reported social anxiety symptoms and a significant improvement in self-esteem at post-treatment as compared to controls. Although not employing a SAD sample specifically, an additional study by Lu et al. (2019) indicated that treatment with MBIs effectively reduced social anxiety symptoms at post-treatment in an 11–13 year old sample. Together, these results provide separate lines of evidence that treatment with MBIs reduces anxiety symptoms in adults; MBIs are effective in reducing anxiety symptoms specifically in adolescents; and that MBIs have the potential to directly engage symptoms that are unique to SAD, particularly along dimensions of positive affect and approach-related emotions, which are known to be significantly diminished in SAD samples (Brown et al., 1998; Kashdan, 2007).

## Feasibility and Acceptability of MBIs for Adolescents

In order for MBIs to be considered a viable treatment option, feasibility and acceptability are critical factors to consider. There is increasing evidence related to the feasibility, acceptability, and positive outcomes for MBIs in the treatment of adolescent psychopathology across a broad range of psychiatric disorders including anxiety. For example, using their “Taming the Adolescent Mind” program, Tan and Martin (2013) conducted a small preliminary intervention study with 10 adolescents with heterogeneous psychiatric diagnoses. A 90% completion rate and high levels of participant-rated program satisfaction and program usefulness were reported. In another small feasibility study (*N* = 11), Ames et al. (2014) delivered an 8-week MBCT intervention for adolescents presenting with residual depression symptoms following standard psychological treatment for a mood or anxiety disorder. Although these participants had not responded to prior psychotherapy, the subsequent MBI was found to have acceptable levels of completion, with seven of the 11 enrolled participants completing the program (two leaving due to relapse, one due to a family situation, and one leaving for undisclosed reasons). Furthermore, participants appeared to find the intervention acceptable, reporting that they had favorable evaluations of MBCT and enjoyed the intervention.

Of particular interest, a small number of studies have examined acceptability and feasibility of MBIs in adolescent anxiety specifically. A qualitative study (*N* = 28) by Van Vliet et al. (2017) examining the impact of an 8-week MBSR intervention for adolescents in a psychiatric residential facility (39% with a primary anxiety disorder diagnosis) found high levels of favorable subjective impressions of the intervention. From post-intervention interviews with adolescents, six themes emerged from the qualitative data: improved mood, enhanced relationship to self, increased self-control, improved problem solving, awareness of the present, and enhanced interpersonal relationships. At the initial post-intervention interview, 75–93% of participants endorsed each theme, although this fell to 50–79% endorsement for each theme at a 3-month follow up interview, potentially suggesting the need for “booster” sessions as a strategy for maintaining treatment gains.

In addition to high levels of feasibility and acceptability for MBIs in anxious adolescent populations, these interventions have not been associated with iatrogenic harm (for meta-analysis see Zoogman et al., 2015). Furthermore, acceptability and feasibility for MBIs have been demonstrated across a variety of populations including anxious children as young as 7–8 years of age (Semple et al., 2005), healthy and sub-clinical adolescent populations (Bluth et al., 2015; Johnson et al., 2016; Bluth and Eisenlohr-Moul, 2017), as well as pre-adolescent and minority and low-income adolescent populations (Liehr and Diaz, 2010; Sibinga et al., 2011). Altogether, the available evidence indicates that MBIs are feasible and acceptable in an anxious adolescent population. Further, this evidence suggests that implementation of MBIs in anxious adolescent samples may hold particular promise for increasing the likelihood of treatment completion, and thus may improve treatment gains.

## Conclusion

Our focus on MBIs for adolescent social anxiety in this brief review is premised upon prior work highlighting it as a period of heightened SAD-relevant risk (given that the mean age of onset falls within this developmental period); relatively better rates of reduction in SAD and remission in comparison to other CBT approaches; and a separate body of work demonstrating the potential for MBIs to directly engage hypothesized maintaining factors of SAD in adult samples. However, there are a few noteworthy weaknesses among these studies that will need to be addressed in future research in order to make more concrete claims regarding the potential efficacy of MBIs for this population. First, the overall lack of research on MBIs for adolescent SAD is clearly the largest limitation. However, the lack of comparison groups across studies currently precludes the possibility of determining whether MBIs may be more efficacious than other treatments in this sector of the population. Therefore, future research should include meaningful comparison groups such as CBT or CGBT in order to more precisely establish efficacy rates between the two treatments in this population. Second, many studies examining MBIs in adolescent populations have utilized small sample sizes, thus limiting more robust conclusions; this should be an active focus in the design of future studies. Finally, the heterogeneity of anxiety disorder diagnoses within the samples in many of the studies involving MBIs may contribute to variable outcomes. In future studies, the focus should be on detailed clinical characterization on as many relevant baseline variables as possible, potentially in single disorder categories such as SAD, which will facilitate future comparisons with a well-characterized comparison group. Despite these weaknesses, and in light of this prior work on MBIs, a unique convergence of factors emerge that suggest the potential efficacy of MBIs for treating socially anxious adolescents. First, promising evidence primarily stemming from the adult literature suggests that MBIs demonstrate the ability to directly engage symptoms of SAD. Further, early evidence in adolescent populations MBIs also show consistent reduction of anxiety symptoms, including symptoms of social anxiety specifically. Moreover, prior work has suggested that MBI interventions have demonstrated high rates of feasibility and acceptability in anxious adolescent samples. Therefore, we conclude that, in conjunction with promising albeit emergent evidence for efficacy in socially anxious adolescents, it is therefore probable that MBIs are a particularly promising and viable treatment approach for reducing symptoms of social anxiety during adolescence. However, it should be noted that there is still a paucity of empirical evidence on MBIs in adolescent SAD in particular, and given the limitations regarding the current landscape of the literature surrounding this question, further work specifically explicating the impact of MBIs on adolescent SAD outcomes is paramount to make more concrete conclusions regarding the promise of this approach.

## Author Contributions

CC, HS-T, MS, TO, and JR were all involved in the conception, drafting, and revisions of the article. All authors contributed to the article and approved the submitted version.

### Conflict of Interest

The authors declare that the research was conducted in the absence of any commercial or financial relationships that could be construed as a potential conflict of interest.
